# Clinical Effect of Posterior Obliqueligament Repair in Anterior Cruciate Ligament Reconstruction Combined With Medial Collateral Ligament Repair: A Retrospective Comparative Study

**DOI:** 10.1111/jcmm.70690

**Published:** 2025-07-24

**Authors:** Qi Ao, Jindong Chen, Yingguang Xie, Yonghua Wang, Xiaoxi Wang, Feng Liu, Zhigang Bai, Shuaifei Zhao, Xiaoyan Du, Liang Zhang, Xiaoyu Gao, Xin Suyalatu

**Affiliations:** ^1^ Ordos School of Clinical Medicine Orthopedics Department Inner Mongolia Medical University Ordos Inner Mongolia China; ^2^ Orthopedics Department Tengzhou Central People's Hospital Tengzhou Shandong China; ^3^ Orthopedics Department Ordos Central Hospital Ordos Inner Mongolia China; ^4^ Neurology Department Dongsheng District People's Hospital Ordos Inner Mongolia China

**Keywords:** anterior cruciate ligament, anteromedial rotatory instability, knee joint, medial collateral ligament, minimally invasive surgery

## Abstract

The stability of the knee joint following anterior cruciate ligament (ACL) restoration combined with medial collateral ligament (MCL) injury repair is crucial. It is yet unknown how posterior oblique ligament (POL) suturing functions in these procedures. This study aimed to assess the impact of POL repair on knee joint stability in patients undergoing ACL reconstruction with MCL repair. We hypothesised that POL repair would further enhance knee joint stability compared to conservative treatment in patients with combined ACL and MCL injuries. A total of 104 patients with ACL and grade III MCL injuries were enrolled and divided into the experimental group (*n* = 53, POL repair) and the control group (*n* = 51, conservative treatment for POL injury). Knee stability was assessed using Ligs Digital Arthrometer (measuring tibial displacement and medial separation) and the Dial test (measuring external tibial rotation). Knee function was evaluated with IKDC, Lysholm, VAS and ROM scores at baseline and 12‐month follow‐up. Both groups showed significant improvements in tibial displacement (ACL SSD) and medial separation (MCL SSD) after surgery compared to baseline (*p* < 0.05). However, intergroup comparisons indicated no statistically significant changes in knee stability metrics (*p* > 0.05). Functional scores (IKDC, Lysholm, ROM and VAS) improved considerably in both groups from baseline, with no significant differences between groups (*p* > 0.05). The Dial test revealed a substantial decrease of external tibial rotation in both groups postoperatively, with no intergroup variations. While ACL reconstruction and MCL repair significantly improved knee joint stability, POL suturing did not have a significant impact on the overall knee stability in patients with ACL and MCL injuries.

## 1 Introduction

With the development of society, people's demand for sports is increasing day by day. Due to the lack of systematic training, weak awareness of sports self‐protection, excessive exercise intensity, or self‐dysplasia and other problems, the number of sports injury patients is increasing year by year. The Anterior cruciate ligament (ACL) is an important structure to maintain the stability of the knee joint. When the knee joint is extended, the average length of the ACL is 32 mm and the width is 7–12 mm, and it accounts for more than 50% of all knee joint injuries [1, 2]. After ACL injury, its limiting effect will be weakened or lost, resulting in forward instability and rotational instability of the knee joint [3].

The medial stable structure of the knee joint mainly includes the medial collateral ligament (MCL) and the posterior oblique ligament (POL), of which MCL is an important structure to resist the valvaration stress of the knee joint and maintain the rotational stability [4, 5, 6]. From the perspective of anatomical structure and biomechanics, MCL is located on the posteromedial side of the knee joint and is divided into two layers: deep MCL (dMCL) and shallow MCL (sMCL), which together with POL and the posteromedial articular capsule form the posteromedial complex of the knee joint [7]. This structure is the main static stable structure of the medial side of the knee joint, and its injury may lead to the instability of the medial side of the knee joint and increase the risk of ACL injury [8]. At present, some scholars have pointed out that POL is the main structure that restricts knee varion and tibial pronation when knee flexion is 0°–30°, and plays an important role in maintaining the stability of knee pronation [9, 10]. Therefore, the combined injury of MCL‐POL leads to unstable valgus and anteromedial rotatory instability (AMRI) of the knee joint. D'Ambrosi et al. [11] conducted a systematic review on the midterm outcomes, complications and return to sports after MCL and POL reconstruction, demonstrating significant improvements in knee stability but also noting potential complications. Additionally, D'Ambrosi et al. [12] reported a high incidence of ramp lesions and a nonnegligible incidence of anterolateral ligament and POL rupture in acute ACL injuries, emphasising the need for comprehensive assessment and treatment of these injuries. These findings highlight the complexity of knee injuries and the importance of addressing all relevant structures to achieve the best possible outcomes.

In terms of treatment, according to the degree of MCL injury, it can be divided into conservative treatment and surgical treatment. A preliminary consensus has been reached that conservative treatment is preferred for mild MCL injuries [13], whereas surgical treatment is generally recommended for multiligament injuries involving severe tears of the Posteromedial complex (PMC), subacute or chronic eversion instability, and in cases requiring arthroscopic ACL reconstruction. However, for the combined injury of MCL‐POL, the influence of POL repair and reconstruction on the stability of forward rotation of the knee joint remains controversial. Some studies suggest that POL reconstruction may enhance rotational stability, while others report no significant additional benefit compared to MCL repair alone [14, 15]. If the treatment is not proper, it is easy to cause knee valvaration and instability, long‐term chronic pain and secondary arthritis and other complications, and even lead to knee dysfunction [16, 17]. This lack of consensus highlights the need for further clinical evidence to guide treatment decisions.

Therefore, the aim of this study was to evaluate the impact of POL repair on knee joint stability and functional outcomes in patients undergoing ACL reconstruction combined with MCL repair. We hypothesised that POL repair would further enhance knee joint stability and functional recovery compared to conservative treatment of POL injury.

## 2 Materials and Methods

### 2.1 Included Patients and Enrollment Criteria

In this study, patients who underwent surgical treatment and were followed up in Inner Mongolia Ordos City Centre Hospital from June 2018 to June 2023 were selected. All patients included in this study were treated with autologous tendon reconstruction of ACL and superficial and deep suture repair of MCL. Among them, the control group underwent conservative treatment for POL injury, and the experimental group underwent suture repair for POL injury. This study has been approved by the Ethics Committee of the hospital, and all subjects have signed informed consent.

At present, the clinical treatment plans for grade I and grade II medial collateral ligament injuries alone (without combined injuries of other ligaments) are basically consistent. For grade I injuries, symptomatic treatment is sufficient. Patients usually recover to normal after a short period of rest, bandage fixation and ice application. Patients with grade II injury have partial ligament tears. To protect the stability of the remaining ligaments, they should wear a knee joint adjustable angle brace. On the basis of preventing valgus with the brace, partial weight‐bearing should be carried out, and strenuous physical activities should be avoided. The range of motion of the knee joint should not be restricted to ensure the flexion and extension function of the knee joint and prevent serious consequences such as fibrosis and stiffness in the joint cavity.

**FIGURE 1:**
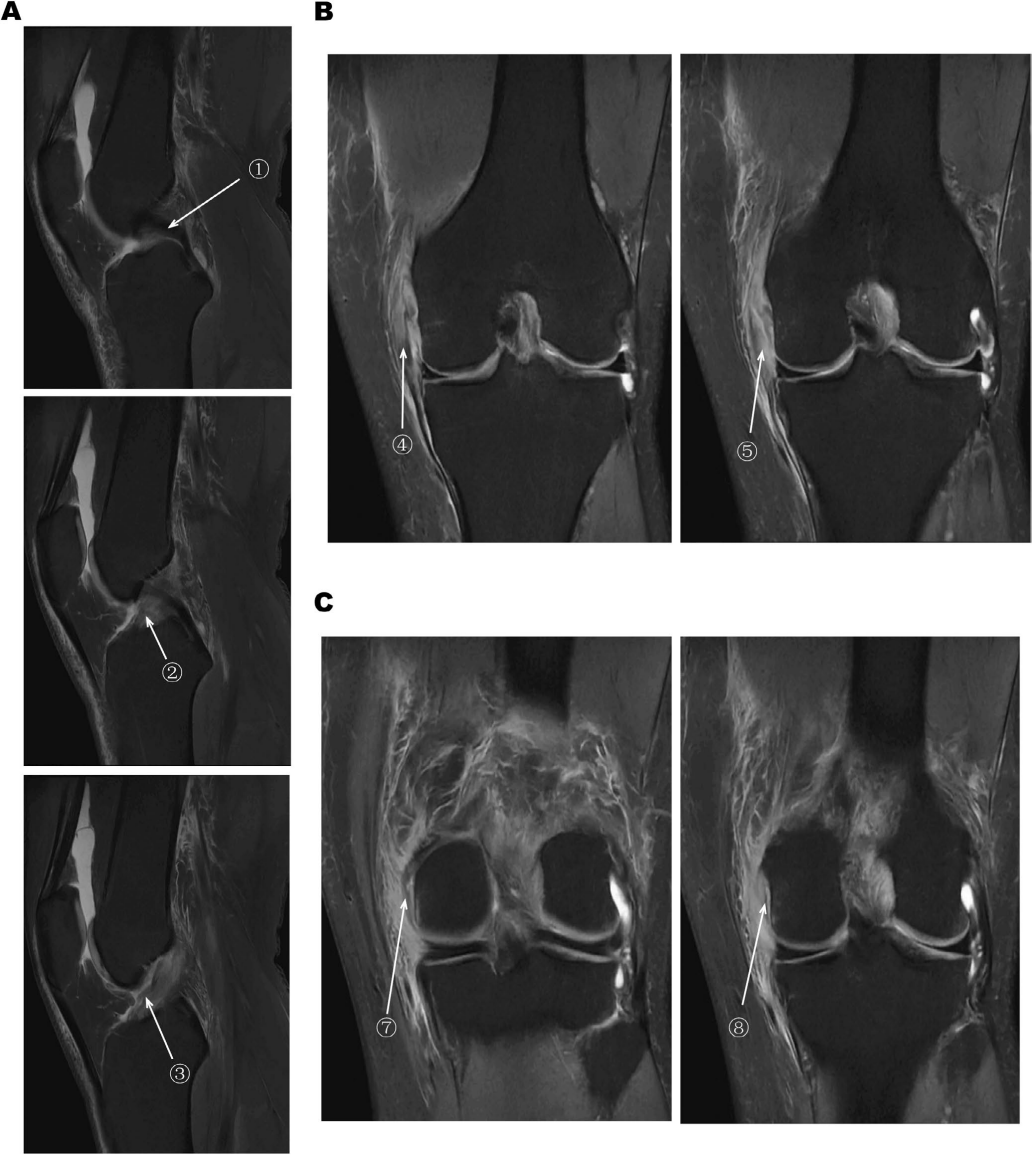
MRI images of knee ligament injury, Makers①–③: The posterior cruciate ligament is running normally with good tension, and the anterior crossover ligament (ACL) is torn. Markers④ and ⑤: Medial collateral ligament (MCL) femoral stop tear. Marker⑥ and ⑦: Posterior oblique ligament (POL) tear.

Screening based on inclusion criteria and exclusion criteria. Inclusion criteria: ① Age 18–65 years old; ② Preoperative physical examination revealed that the lateral stress test of the knee joint, the anterior drawer test and the Lachman test were positive. ③ The results of imaging examinations showed: ACL fracture combined with grade III MCL injury; ④ It is agreed that autologous single‐bundle reconstruction of the thin femoral tendon, semitendinotendon and anterior cruciate ligament, as well as superficial and deep suture repair, will be performed during the acute stage, with the time interval between injury and surgery not exceeding 3 weeks. ⑤ The patient had unilateral knee injury, while the contralateral knee was diagnosed as normal by magnetic resonance examination. Exclusion criteria: ① Open knee joint injury, knee joint infection; ② Combined with posterior cruciate ligament, posterolateral complex, or grade III medial collateral ligament injury. Injuries to other ligaments of the knee joint. ③ Patients with knee joint bone structure injuries such as severe degenerative changes in articular cartilage (Outerbridge grade III or above) and neurovascular injuries such as synovitis and rheumatoid arthritis; ④ Severe dysplasia of the force line in the lower extremities. ⑤ Patients who cannot tolerate preoperative procedures (Figure 1).

### 2.2 Perioperative Operation

All procedures are performed by a team of experienced orthopaedic surgeons. The procedure was performed under subarachnoid anaesthesia combined with continuous epidural anaesthesia. The patient was placed in a supine position and a tourniquet was applied to the upper third of the femur of the affected limb. The stability of the knee joint was examined and confirmed under anaesthesia. Lachman test, valgus stress test and Slocum test were positive in all patients. The anterior medial and anterolateral approaches of the knee joint were adopted to examine the cruciate ligament injury of the knee joint under arthroscopy, and the corresponding lesions were treated.

The procedure is divided into three parts. ① ACL reconstruction: using a single bundle of four strands autologous gracilis tendon and semitendinosus tendon reconstruction. Graft acquisition and preparation: A 4 cm medial tibial incision was made about 4 cm away from the joint line and at the medial tibial tubercle. Subcutaneous separation was performed to reveal the anseris foot stop. A short incision was made along the upper margin of sartorial muscle to reveal the gracilis tendon and semitendinosus tendon, taking care not to damage the dMCL. Use tissue scissors to release the tendon, release the tendon to the fibrous extension of the gastrocnemius and semimembrane muscle, and completely release the accessory tendon. The tendon was separated from the subperiosteal to the tibial stop, then cut sharply, and the thin semitendinous tendon was completely removed with a closed tendinous extractor. After the muscle tissue was removed from the tendon, the end of the tendon was braided with ligamentous suture, and two tendons were folded in half to form four strands. The diameter of the tendon was measured to be 8 mm.

Establishment of femoral tunnel: Lumbar puncture needle was used to determine the optimal position of the low medial approach, the stump of ACL and the resident's ridge were used to locate the femoral tunnel, and radiofrequency labeling was used. The offset guide was used to bend the knee 120° to drill into the guide needle, a 4.5 mm hollow drill was used to drill through the lateral condylar cortex of the femur through the guide needle, and the length of the bone tunnel was measured with an 8 mm hollow drill to prepare the thick bone canal of the femur. The posterior wall of the femoral tunnel remains 2 mm. After the debris in the bone tunnel is cleaned up with the planer knife, the guide needle with small hole is inserted into the guide line of the belt coil, and the guide needle is inserted through the thigh lateral skin through the femoral tunnel. Establishment of the tibial tunnel: The tibial tunnel was located by using the ACL tibial locator with reference to the tibial stump of the ACL, the anterior angle of the lateral meniscus, the medial intercondylar ridge of the tibia, and the posterior cruciate ligament under arthroscopy. The guide needle was drilled into the knee 10 mm in the direction of 50°–55° towards the articular surface of the tibia, and the tibial canal was prepared by using an 8 mm hollow drill after the impact of the top of the non‐intercondylar fossa in the flexion and extension test.

② sMCL and dMCL suture: A 4–6 cm incision was made from the medial femoral condyle of the knee joint to the upper part of the anseropodium of the tibia to gradually reveal the medial structure of the knee joint and confirm the location of the MCL tear. When the dMCL or sMCL tear is located at the femoral or tibial impingement, the suture repair and impingement reconstruction are performed using 1–2 anchors with wire. When in the body, the suture is performed with the Axicon 2 suture. The medial articular capsule is repaired at the same time. The flexion and extension knee joint is sutured to test the suture tension.

**FIGURE 2:**
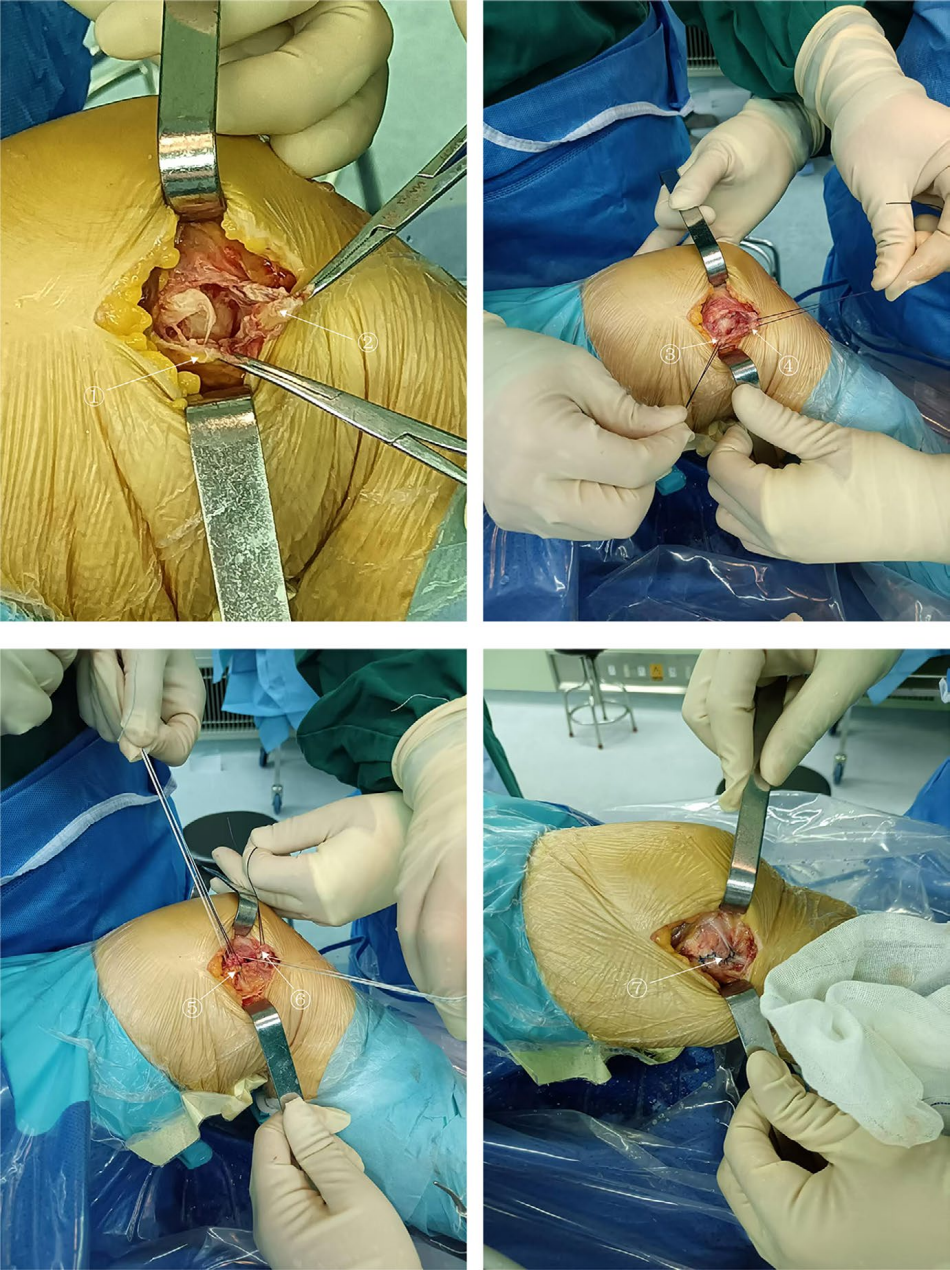
Intraoperative pictures of the autologous tendon reconstruction of ACL and superficial and deep suture repair of MCL. Marker①: POL tear stump, Marker②: MCL tear stump, Marker③ and ⑤: POL repair, Marker④ and ⑥: MCL repair, Marker⑦: MCL and POL suture repair.

③ POL suture in the experimental group: the posteromedia structure of the knee joint was revealed, and the sMCL and dMCL structures were distinguished. POL was the posteromedia structure but not the medial structure, and it was an approximately triangular structure. After confirmation of POL damage, the avulsion injury at the stop was repaired with 1–2 anchor stitches. If the body was torn, the avulsion injury was repaired with Aixikang No. 2 suture. Conservative treatment group: posterior oblique ligament injury was repaired without suture. Close the incision layer by layer and wrap with an elastic bandage (Figure 2).

After the recovery from anaesthesia, we performed the isometric contraction of the quadriceps muscle with hinge support at 0° and ankle pump exercises of both lower limbs. Within 1 week after surgery, under the guidance of the rehabilitator, we got down from the bed and bent the knee to 90°. Within 2 weeks, we got down to the ground to support the two crutches and fixed the hinged support to 0° for the affected limb to avoid weight bearing. For 2–6 weeks, we performed straight leg elevation exercises beside the bed, while walking with partial weight under the protection of fixed double crutches at 30° to 60°. 6–8 weeks after surgery, single crutches were changed, most of the weight‐bearing walking was carried out under the protection of 90° support, and resistance exercises of 2 to 5 kg of quadriceps muscle and straight leg elevation exercises were added. After 8 weeks, the crutches were gradually removed and full weight‐bearing walking was carried out. The hinged brace was worn for 3 months after surgery, and daily exercise such as jogging began gradually at 6 months after surgery.

### 2.3 Clinical Data Collection

**FIGURE 3:**
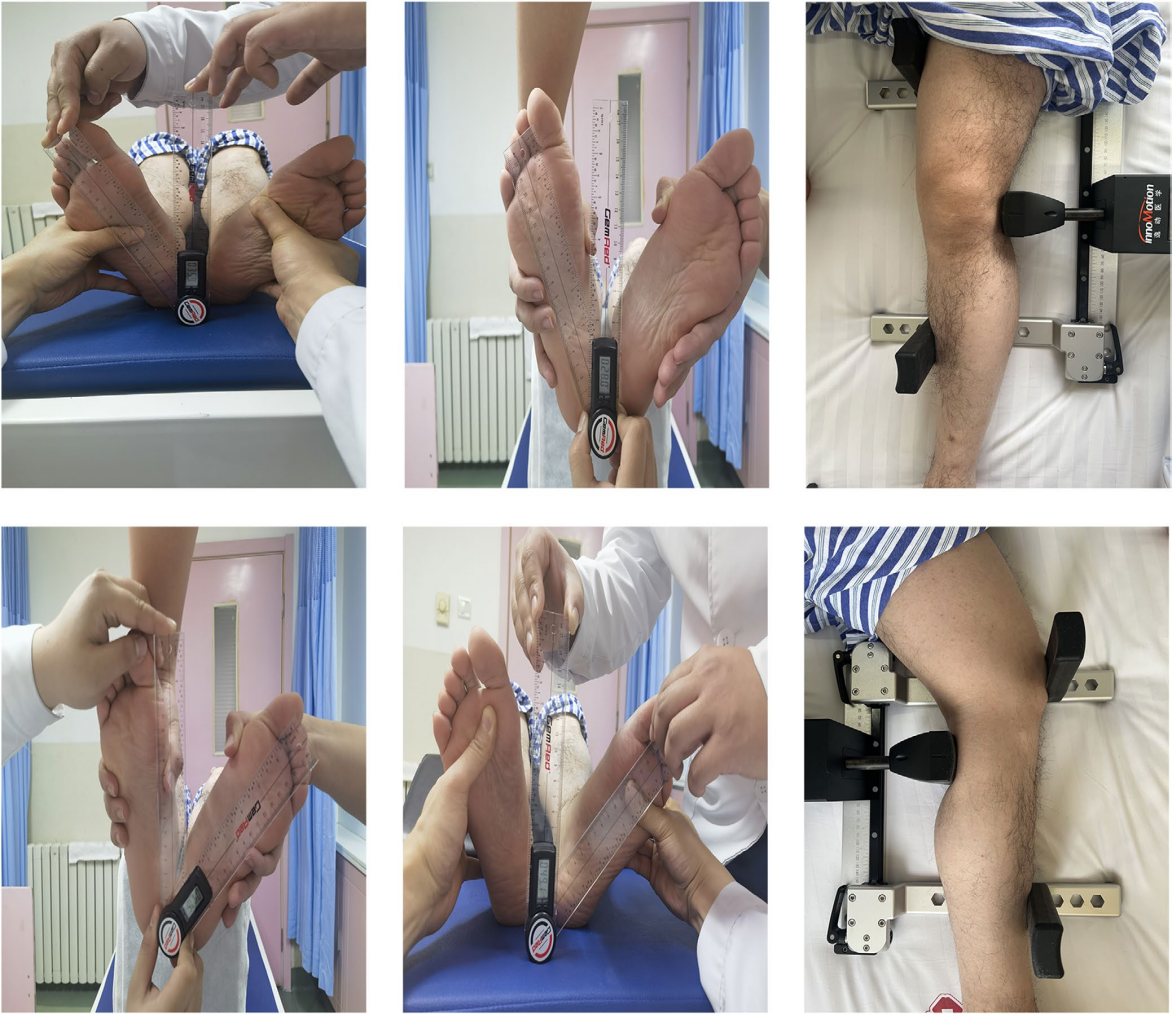
In the Dial test, the deflection angle was measured with a high precision display ruler, and the relaxation of MCL and ACL was measured with Ligs Digital Arthrometer.

Clinical history data of the two groups were included. The absolute value of SSD of relative displacement of the knee joint under different stresses was measured by Ligs Digital Arthrometer before operation and at the last follow‐up in both groups. The lateral lying position was taken to measure the forward relaxation of the patient when the knee was bent at 30°. The main push rod was aligned with the patient's tibial tubercle to make the patient's patella close to the groove of the L‐type limiting assembly. At the same time, the lower support assembly was adjusted to the farthest end and the lower leg segment was fixed with the I‐type limiting assembly, and the forward stress was applied to the tibia. When the pressure values of 90 N, 120 N and 150 N were recorded respectively, the corresponding forward relative displacement of the tibia was obtained. The internal relaxation of the patient was measured in the supine position when the knee joint was extended. The main push rod was aligned with the lateral space of the patient's knee joint, and the lower femur and lower tibia were fixed with type I limiting components to form the knee joint varus stress. The relative displacement of the medial opening of the knee joint was recorded when the pressure values were 60 N, 90 N and 120 N, respectively. The external tibial rotation test (Dial test) was used to measure the external tibial rotation angle of the two groups before and at the last follow‐up. When the knees were bent at 30° and 90°, the vertical line of the ground was taken as the central axis, and the degree of external rotation of the knees was compared with a high‐precision digital angle scale. Each data point was measured three times and averaged. After each test, the knee joint was manually restored (Figure 3).

Lyshlom function score, IKDC function score, range of motion ROM and VAS pain score were used to evaluate knee function. The last follow‐up time of all patients was more than 12 months from the time of operation, and the knee joint function met the needs of daily life under the guidance. The knee joint incision healed in stage I after surgery, and no complications such as knee joint infection, knee joint stiffness and ligament re‐rupture occurred.

## 3 Statistical Analysis

SPSS 29.0 software and R software version 4.0.3 were used for statistical analysis. All tests were double‐sided and the test level was set to 0.05. Descriptive statistics summarised the treatment groups and overall demographic data and baseline characteristics. Demographic data and other baseline characteristics will be compared using T‐tests, Chi‐square tests, or rank sum tests to measure group parity. Changes in VAS scores from baseline were compared between the two groups for primary and secondary indicators, and multivariate covariance analysis was performed by adjusting for sex and age, BMI and previous disease history. Visualise the data using R software version 4.0.3.

## 4 Power Analysis Performed

In this study, the IKDC index was used for power analysis. The mean changes of the two groups were 37.01 and 36.50 respectively, and the standard deviations were 0.67 and 0.66. The included samples of the two groups were 53 and 51. The actual data analysis showed that the power was 97.2%, exceeding the general power requirement of 80% and meeting the requirements (Figure S1).

## 5 Results

### 5.1 Baseline Characteristics of Patients Control Group and the Experimental Group

A total of 104 patients were enrolled in this study and completed the 12‐month follow‐up according to the inclusion and exclusion criteria. Fifty‐three patients made up the experimental group. Another 51 patients were included as the control group. There were no significant differences in baseline data including gender, age, height, weight, BMI, clinical history and length of stay between the two groups (*p* > 0.05). The results show that the grouping is valid and can be used for further study (Table 1).

**TABLE 1:** The baseline data of the two groups.

	Total (*N* = 104)	Group 1 (*N* = 53)	Group 2 (*N* = 51)	*p*
Sex				0.726
Female	67 (64.4%)	35 (66.0%)	32 (62.7%)	
Male	37 (35.6%)	18 (34.0%)	19 (37.3%)	
Age	42.14 (12.73)	40.58 (14.30)	43.76 (10.77)	0.318
Height (cm)	171.14 (5.99)	170.77 (5.86)	171.53 (6.15)	0.451
Weight (kg)	70.21 (7.04)	70.47 (6.74)	69.94 (7.39)	0.722
BMI (m/kg^2^)	24.06 (2.59)	24.20 (2.48)	23.91 (2.71)	0.886
Trauma (time)	1.52 (1.09)	1.55 (1.05)	1.49 (1.14)	0.613
Smoking	57 (54.8%)	32 (60.4%)	25 (49.0%)	0.245
Hypertension	45 (43.3%)	24 (45.3%)	21 (41.2%)	0.673
Diabetes	20 (19.2%)	11 (20.8%)	9 (17.6%)	0.688
Copd	7 (6.7%)	3 (5.7%)	4 (7.8%)	0.713
Number of days in hospital	14.79 (8.61)	14.60 (7.36)	14.98 (9.81)	0.958

### 5.2 The Difference of Knee Ioint SSD Between Each Group Before Surgery and the Last Follow‐Up and Between the Two Groups Were Compared

**FIGURE 4:**
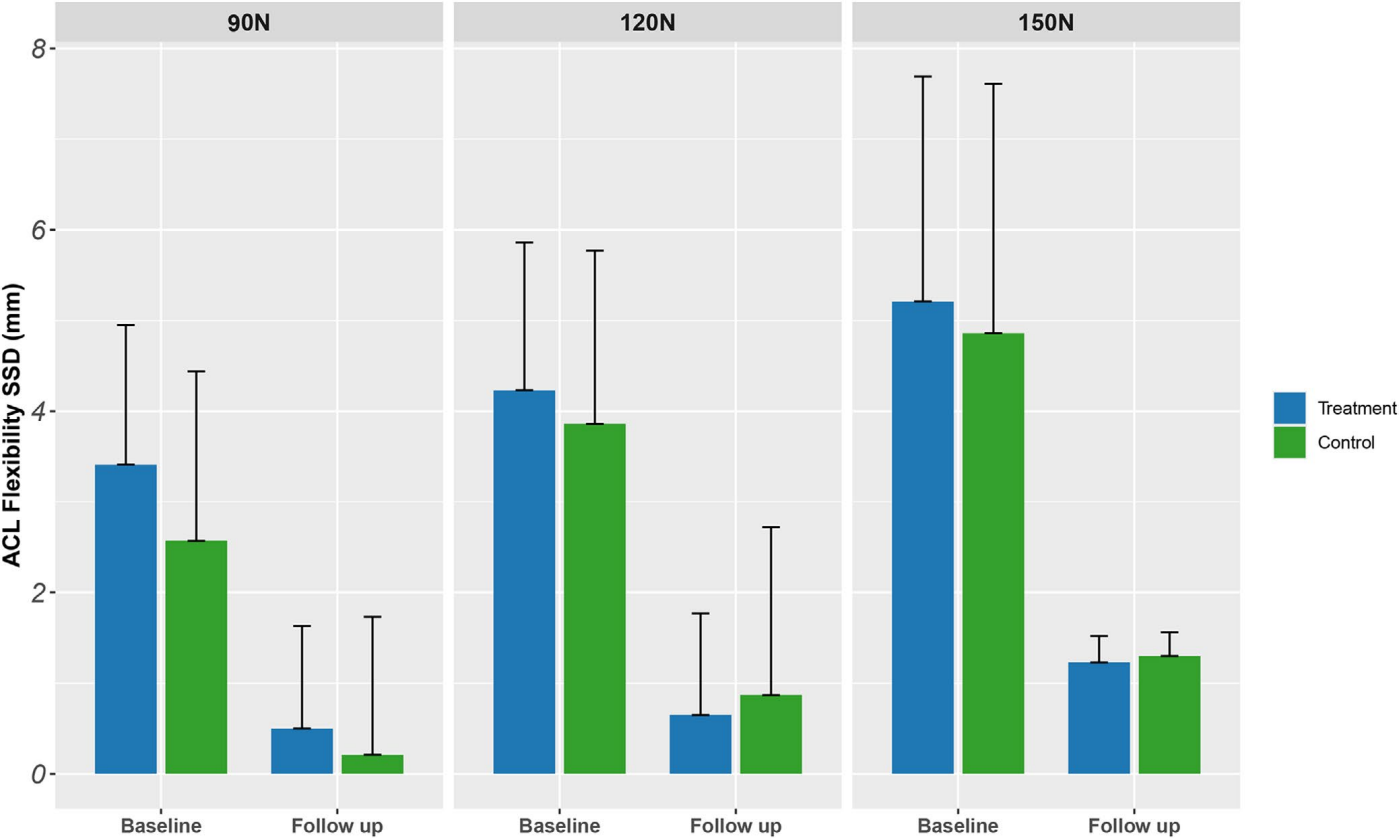
The forward relaxation SSD of ACL.

**FIGURE 5:**
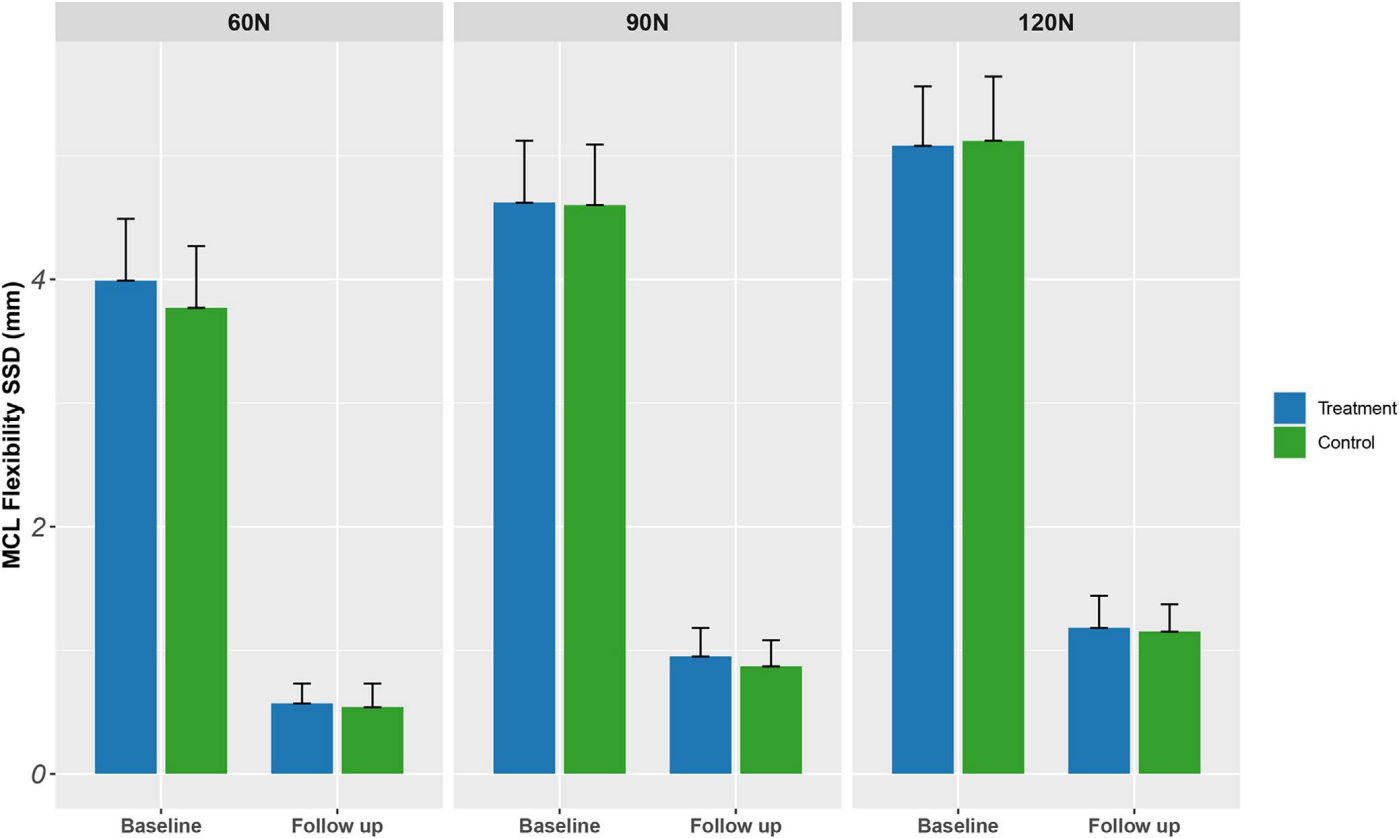
The inward relaxation SSD of MCL.

All patients were able to tolerate stress loads when they were applied with Ligs Digital Arthrometer. The results showed that at the last follow‐up, both the forward relaxation and inward relaxation SSD of ACL and MCL measured in the two groups were significantly reduced compared with those before surgery, and the difference was statistically significant (based on change from baseline), as shown in Table 2. Before surgery and at the last follow‐up, inter‐group comparison showed no significant difference between forward and inward relaxation SSD (*p* > 0.05) (Table 2). The resulting results are shown in Figures 4 and 5.

**TABLE 2:** The difference of knee joint SSD between each group before surgery and the last follow‐up and between the two groups was compared.

Outcome	*N*	Visit	Group 1 (*N* = 53)		Group 2 (*N* = 51)		Difference between groups in change from baseline	*p*
			Mean score	Change from baseline	Mean score	Change from baseline		
ACL (mm)	90 N	Baseline	3.41 (1.54)		2.57 (1.87)			
		Follow up	0.50 (1.13)	−1.84 (−2.45, −1.23)	0.21 (1.52)	−2.29 (−2.90, −1.68)	0.45 (−0.08, 0.99)	0.0980
	120 N	Baseline	4.23 (1.63)		3.86 (1.91)			
		Follow up	0.65 (1.12)	−3.43 (−4.06, −2.79)	0.87 (1.85)	−3.52 (−4.15, −2.89)	0.09 (−0.45, 0.64)	0.7331
	150 N	Baseline	5.21 (2.48)		4.86 (2.75)			
		Follow up	1.23 (0.29)	−3.81 (−3.94, −3.68)	1.30 (0.26)	−3.74 (−3.87, −3.61)	−0.07 (−0.18, 0.04)	0.2094
MCL (mm)	60 N	Baseline	3.99 (0.50)		3.77 (0.50)			
		Follow up	0.57 (0.16)	−3.34 (−3.41, −3.27)	0.54 (0.19)	−3.33 (−3.41, −3.26)	−0.01 (−0.07, 0.06)	0.8645
	90 N	Baseline	4.62 (0.50)		4.60 (0.49)			
		Follow up	0.95 (0.23)	−3.65 (−3.75, −3.55)	0.87 (0.21)	−3.73 (−3.83, −3.63)	0.07 (−0.01, 0.16)	0.0995
	120 N	Baseline	5.08 (0.48)		5.12 (0.52)			
		Follow up	1.18 (0.26)	−3.94 (−4.04, −3.83)	1.15 (0.22)	−3.96 (−4.06, −3.85)	0.02 (−0.07, 0.11)	0.6678

### 5.3 Comparison of Knee Joint Function Between the Two Groups Before Surgery and the Last Follow‐Up

**FIGURE 6:**
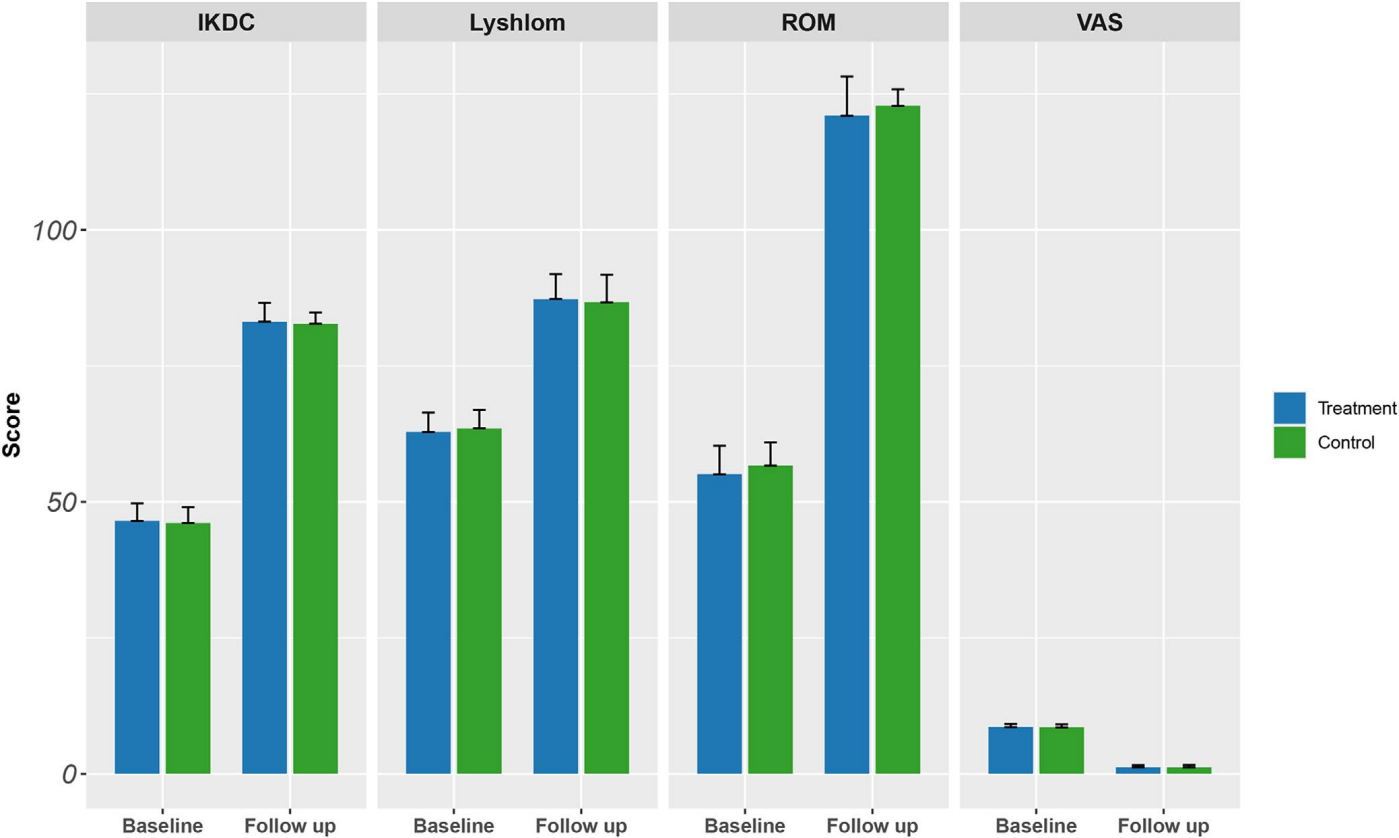
Comparison of knee joint functional scores among different surgical methods.

IKDC score, Lyshlom score, VAS score and ROM of knee joint of patients in both groups were significantly improved compared with those before surgery at the last follow‐up, and the differences were statistically significant (data based on change from baseline). However, there was no statistically significant difference between the two groups of patients (*p* > 0.05), as shown in Table 3, which was also shown in Figure 6.

**TABLE 3:** Comparison of knee joint function between and within the two groups.

Outcome	Visit	Group 1 (*N* = 53)		Group 2 (*N* = 51)		Difference Between Groups in Change from baseline	*p*
		Mean score	Change from baseline	Mean score	Change from baseline		
IKDC	Baseline	46.49 (3.21)		46.10 (2.91)			
	Follow up	83.13 (3.46)	37.01 (35.69, 38.33)	82.75 (2.10)	36.50 (35.18, 37.81)	0.51 (−0.63, 1.65)	0.3731
Lyshlom	Baseline	62.85 (3.56)		63.51 (3.40)			
	Follow up	87.30 (4.61)	25.11 (22.98, 27.24)	86.71 (5.04)	24.39 (22.28, 26.49)	0.72 (−1.08, 2.52)	0.4262
ROM	Baseline	55.08 (5.26)		56.67 (4.26)			
	Follow up	121.00 (7.19)	65.06 (62.69, 67.42)	122.81 (3.06)	66.19 (63.84, 68.55)	−1.14 (−3.19, 0.92)	0.2747
VAS	Baseline	8.61 (0.56)		8.56 (0.53)			
	Follow up	1.21 (0.39)	−7.26 (−7.43, −7.09)	1.20 (0.42)	−7.28 (−7.46, −7.11)	0.03 (−0.12, 0.18)	0.7343

### 5.4 The Dial Test was used to describe the external rotation angle of the tibia and the results were analysed

**FIGURE 7:**
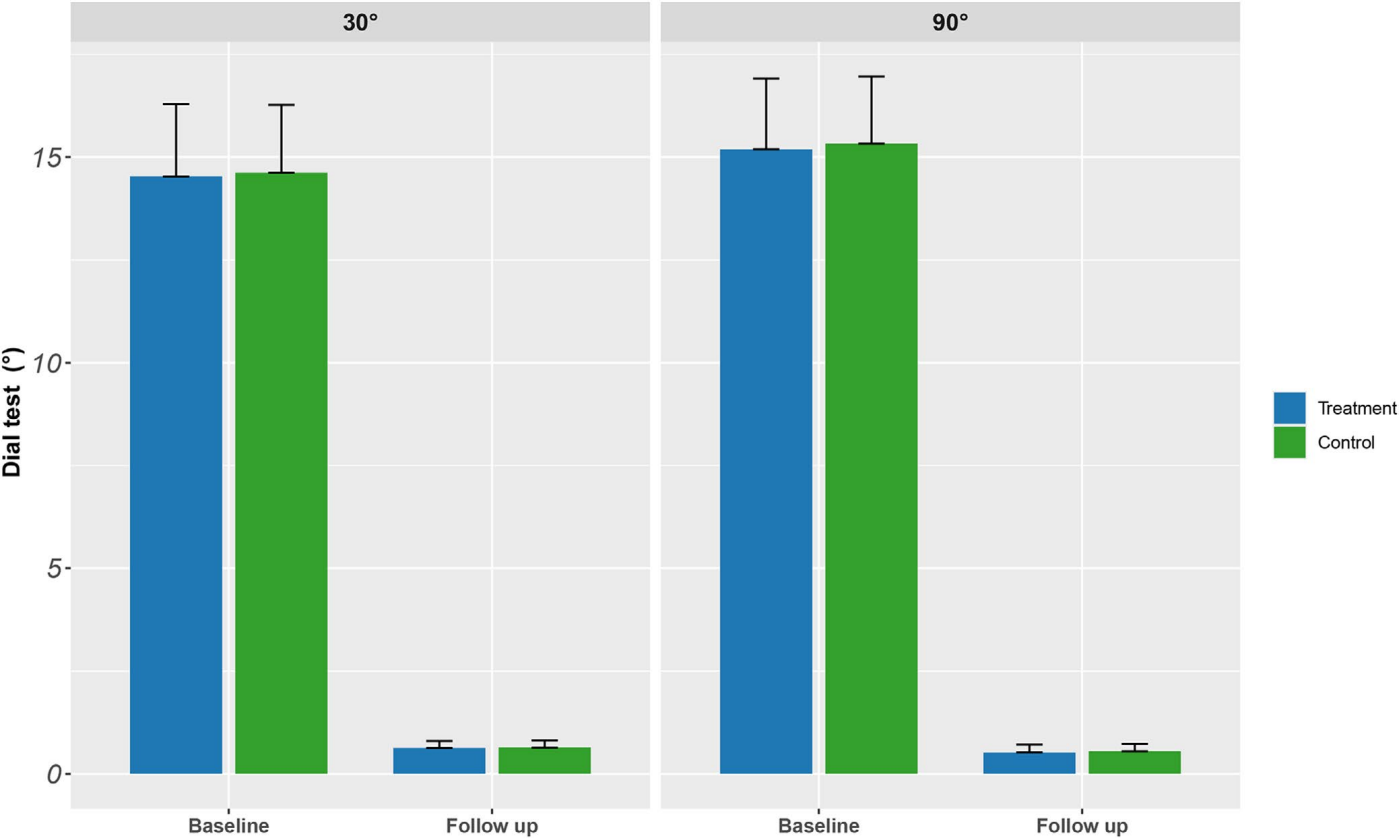
Dial test measures the external rotation angle of the tibia.

All patients could accept Dial test. The results showed that at the last follow‐up, the external rotation angle of the tibia in the two groups was lower than before surgery, and the difference was statistically significant (based on baseline changes). Before surgery and at the last follow‐up, there was no significant difference between groups (*p* > 0.05) (Table 4). The result is shown in Figure 7.

**TABLE 4:** The dial test.

Outcome	Visit	Group 1 (*N* = 53)		Group 2 (*N* = 51)		Difference between groups in change from baseline	*p*
		Mean score	Change from baseline	Mean score	Change from baseline		
Flexion(30°)	Baseline	14.53 (1.76)		14.62 (1.65)			
	Follow up	0.63 (0.17)	−13.95 (−14.00, −13.90)	0.64 (0.17)	−13.95 (−14.00, −13.90)	−0.00 (−0.05, 0.04)	0.9169
Flexion(90°)	Baseline	15.19 (1.72)		15.33 (1.63)			
	Follow up	0.52 (0.19)	−14.76 (−14.83, −14.69)	0.55 (0.18)	−14.74 (−14.81, −14.67)	−0.02 (−0.08, 0.05)	0.6053

### 5.5 Comparison of Surgical Complications Between the Two Groups of Patients

Two patients underwent revision surgery due to chronic medial instability, and four patients underwent revision surgery of the anterior cruciate ligament due to anterior and posterior instability. All these six patients were from the repair group. One patient in the reconstruction group underwent knee release surgery due to flexion problems. In a patient of the reconstruction group, the MCL failed to grow into the femur due to the removal of screws from the femoral tunnel 3 months after the operation. During the follow‐up period, no complications such as graft rejection, medial numbness, or infection were found.

## 6 Discussion

Our study aimed to evaluate the impact of POL repair on knee joint stability in patients undergoing ACL reconstruction combined with MCL repair. The primary hypothesis was that POL repair would further enhance knee joint stability compared to conservative treatment. However, our results indicate that while ACL reconstruction and MCL repair significantly improved knee joint stability, POL repair did not have a significant impact on overall knee stability in patients with combined ACL and MCL injuries.

The reconstruction of ACL fracture has become the most commonly used treatment, and the main goal of this plan is to allow patients to receive physical rehabilitation treatment as soon as possible to restore function and restore movement to the pre‐injury level as soon as possible, without exposing patients to inappropriate risk of re‐injury [18, 19, 20, 21]. AMRI is an injury caused by excessive external rotation of the knee joint, which leads to pathological forward subluxation of the medial tibial plateau relative to the medial condyle of the femur, accompanied by ACL injury or sMCL tear, and is an indication for surgical repair of MCL with or without POL [22]. This study utilised single‐bundle ACL reconstruction and MCL repair techniques to address AMRI. Postoperatively, Lachman and valgus stress tests were negative in all patients, indicating successful restoration of knee stability. However, two patients with sMCL body tears still showed positive Slocum tests, suggesting that further evaluation and potential additional treatment may be necessary in such cases.

The functional outcomes of the patients were evaluated using the IKDC score, Lysholm score, VAS for pain and ROM. Both the experimental and control groups showed significant improvements in these metrics at the last follow‐up compared to preoperative values. However, there were no statistically significant differences between the two groups, indicating that POL repair did not provide additional benefits in terms of functional recovery.

The biomechanical role of the POL in knee stability has been a subject of debate. Some studies suggest that POL repair can enhance rotational stability, while others report no significant additional benefit compared to MCL repair alone. Studies have shown [23] that isolated POL damage is rare. In 99% of grade III MCL injuries, POL damage is present, including focal injury (70.6%) and scattered injury (29.4%), and meniscus or cruciate ligament injury is present in 78% of cases. Simultaneous reconstruction of sMCL and POL can improve knee stability and biomechanics. Rayes et al. [24] showed that valvaration relaxation despite full extension of the knee after ACL reconstruction was the result of POL injury and PMC instability, and this result may lead to increased ACL graft tension when the knee is bearing weight. The study also pointed out that POL repair is easier when the knee is in 90° flexion. Stress tests must be performed during stretching. Kerzner et al. [25] used Achilles tendon allografts to reconstruct MCL and POL during knee arthroplasty, and the results showed that MCL and POL double‐strand reconstruction can improve the stability of valvaration after knee arthroplasty and enhance the stability of the posterior joint capsule.

MCL mainly stabilises the valgus stress of the knee joint and is most effective when the knee joint flexion is 60°. sMCL also acts as a secondary stabiliser on the sagittal plane and as a constraint on the anterogradic translation of the tibia. When the knee joint is extended, dMCL is an auxiliary valgus stabilisation device. POL is an important constraint on valgus and pronation in extension and 0°–30° flexion angles [26]. Isolated injury of sMCL and combined lesions of sMCL and dMCL can lead to excessive relaxation of knee joint valvaration and anterior medial tibial rotation [27]. Some studies have found that the role of the posterior oblique ligament in controlling AMRI can be negligible; POL is the main constraint of tibial internal rotation and the secondary constraint of knee ectropion rotation. MCL has the greatest effect on limiting excessive knee ectropion rotation in the range of 0°–120°, and it has the greatest effect on resisting excessive external rotation in the range of 30°–120°. The posterior oblique ligament restricts excessive pronation only when the knee is extended and flexes by 30° [28, 29]. In recent years, there have been studies comparing POL repair and conservative treatment for patients with ACL combined with MCL injury, and KT‐1000 was used to measure forward stability. The research results showed that there was no significant difference between the postoperative and repair effects of POL conservative treatment [30].

In this study, the anteromedial relaxation of the knee joint was measured using the Ligs Digital Arthrometer, and the Dial test was employed for the assessment of knee joint injuries. As a new type of auxiliary examination instrument, Ligs Digital Arthrometer (Shanghai Yidao Medical Technology Co. LTD.), Model: DPE‐LAL was used to measure the forward relaxation of bilateral knee joints under different stresses, calculate the side‐to‐side difference (SSD) of the tibia on both sides, and measure the forward relative displacement between the tibia and femur under different stresses. In recent years, it has been reported that its effect is similar to that of KT‐1000 and other commonly used clinical knee joint forward instability test instruments, and the measurement results are more sensitive under 30 N and 60 N stresses, while the measurement results are highly sensitive under 90 N, 120 N and 150 N stresses [31, 32]. In addition, the device can also apply knee valgus stress to the patient's knee joint and measure the medial knee joint relaxation by calculating the SSD value of the medial knee joint separation degree. Therefore, in this study, a stress of 90 N, 120 N and 150 N was applied to measure the forward relaxation of ACL. Since MCL is less affected by muscle and fat, and the medial pain of patients after injury is more obvious, some patients cannot tolerate 150 N valgus stress. Therefore, the internal relaxation of MCL is measured by applying 60 N, 90 N and 120 N stresses.

In this study, the anterio‐inward relaxation of the knee joint was measured by Ligs Digital Arthrometer SSD value increased with the increase of stress, and a large relaxation ratio was observed at 150 N stress load. Under maximum stress, the SSD values of ACL forward relaxation and MCL inward relaxation in both the experimental group and the control group changed significantly before surgery and at the last follow‐up, indicating that surgical intervention has a significant effect on SSD values of ACL forward inward relaxation. However, the comparison of SSD values between patients in the experiment and those in the control group showed no statistical significance, indicating that ACL reconstruction and MCL repair can basically meet the daily needs of patients with intraoperative POL suture or not, ACL combined with MCL‐POL injury.

A number of scholars have demonstrated that the Dial test, modified by the tibiofemoral rotation test, is used to evaluate abnormal external rotation of the tibia, and can be used to distinguish between simple posterior external complex injury and posterior cruciate ligament combined external complex injury [33, 34, 35]. The Dial test is performed in a supine position, starting with a knee bend of 30°, with both hands gripping the patient's feet, holding the heel, placing the thumb on the medial edge of the foot, and holding the outside and heel of the foot with four fingers, while applying maximum external rotation force, assessing the foot‐thigh angle and comparing it with the contralateral. Then bend the knees to 90° and measure the external rotation angle again. During the examination, the relative position of the tibial tubercle and the femoral condyle is touched to determine the degree of external rotation of the tibial tubercle relative to the femoral condyle after the application of external rotation stress, which is helpful to evaluate the external rotation angle of the tibia. The measurement took the medial boundary of the foot as the reference line. Dial tests at 30° and 90° knee flexion have important clinical significance for significantly increasing external rotation of grade 3 medial knee injury [22, 36].

Our study results indicate that there are no significant differences in the knee stability indices between the conservative treatment group and the POL repair group. For orthopaedic surgeons, these results suggest that routine POL repair may not be necessary in patients with ACL combined with MCL injuries, provided that ACL reconstruction and MCL repair are performed appropriately. Avoiding unnecessary POL repair can simplify the surgical procedure, reduce operative time, lower surgical trauma and potentially minimise postoperative complications such as stiffness, scar formation, or prolonged rehabilitation. According to Griffith [36], simply severing POL did not significantly increase valgus and rotation angles at all knee flexion angles compared to intact knee flexion angles, so POL had no significant effect in resisting valgus rotation. sMCL provides primary external rotation stability at 30° knee flexion, and sMCL significantly increases knee valvaration when femur arrest tears, but does not significantly increase valvaration when tibial arrest tears, which may be related to the distal division not crossing the joint line, and this observation is of great significance. Although the results of this study indicated no significant advantage of POL repair in the general patient population with ACL and MCL injuries, POL repair may still be considered in specific clinical situations. For instance, patients with high functional demands, such as athletes or individuals involved in frequent pivoting and rotational activities, might benefit from additional rotational stability provided by POL repair. Furthermore, cases with severe PMC injury, chronic valgus instability, or revision surgeries may warrant POL reconstruction to achieve optimal joint stability and prevent secondary complications. These clinical scenarios should be carefully evaluated to tailor individualised treatment strategies.

This study has several limitations. First, the sample size was relatively small and derived from a single centre, which may limit the generalisability of the findings. Future multicentre studies with larger patient cohorts are necessary to validate these results. Second, due to the retrospective design, potential selection bias and confounding factors cannot be entirely ruled out, despite the use of strict inclusion and exclusion criteria. Third, the study was not a fully randomised controlled trial, and double‐blinding was not implemented, which might have introduced observer bias during clinical assessments. In addition, the relatively short follow‐up period limited the assessment of long‐term stability and functional outcomes after surgery. Further studies with larger sample sizes and extended follow‐up durations are warranted to evaluate the long‐term efficacy and durability of the procedures.

## 7 Conclusions

In the case of ACL combined with MCL‐POL injury, the patient underwent single‐beam ACL reconstruction and deep and shallow MCL suture repair, which significantly improved the anterior medial rotational instability of the patient's knee joint. Ligs Digital Arthrometer measured the anterior tibial displacement and medial separation of the knee joint, Dial test measured the reduction of tibial rotation angle, and the knee joint stability was good at the last follow‐up, which is of great significance for the recovery of knee joint stability. However, the results of this study showed that whether POL was sutured in ACL combined with MCL‐POL had no significant effect on the stability of the knee joint.

## Author Contributions

**Qi Ao:** conceptualization (equal), data curation (equal), funding acquisition (equal), methodology (equal), writing – original draft (equal), writing – review and editing (equal). **Jindong Chen:** conceptualization (equal), data curation (equal), investigation (equal), methodology (equal), resources (equal), software (equal), writing – original draft (equal), writing – review and editing (equal). **Yingguang Xie:** conceptualization (equal), funding acquisition (equal), investigation (equal), methodology (equal), software (equal), writing – original draft (equal), writing – review and editing (equal). **Yonghua Wang:** conceptualization (equal), data curation (equal), formal analysis (equal), methodology (equal), resources (equal), writing – original draft (equal), writing – review and editing (equal). **Xiaoxi Wang:** data curation (equal), formal analysis (equal), funding acquisition (equal), investigation (equal), methodology (equal), writing – original draft (equal), writing – review and editing (equal). **Feng Liu:** formal analysis (equal), funding acquisition (equal), software (equal), supervision (equal), writing – original draft (equal). **Zhigang Bai:** data curation (equal), formal analysis (equal), funding acquisition (equal), resources (equal), writing – original draft (equal), writing – review and editing (equal). **Shuaifei Zhao:** data curation (equal), formal analysis (equal), funding acquisition (equal), investigation (equal), methodology (equal), writing – original draft (equal), writing – review and editing (equal). **Xiaoyan Du:** data curation (equal), funding acquisition (equal), investigation (equal), methodology (equal), software (equal), writing – original draft (equal). **Liang Zhang:** formal analysis (equal), funding acquisition (equal), methodology (equal), writing – original draft (equal), writing – review and editing (equal). **Xiaoyu Gao:** formal analysis (equal), project administration (equal), software (equal), supervision (equal), validation (equal), writing – review and editing (equal). **Xin Suyalatu:** formal analysis (equal), project administration (equal), supervision (equal), validation (equal), writing – review and editing (equal).

## Ethics Statement

The authors have nothing to report.

## Consent

The authors have nothing to report.

## Conflicts of Interest

The authors declare no conflicts of interest.

## Supporting information


Appendix S1.

Appendix S2.

Appendix S3.

## Data Availability

The data that support the findings of this study are available from the corresponding author upon reasonable request.

## References

[1] G. Kakavas , N. Malliaropoulos , G. Bikos , et al., “Periodization in Anterior Cruciate Ligament Rehabilitation: A Novel Framework,” Medical Principles and Practice 30, no. 2 (2021): 101–108. 33264774 10.1159/000511228 PMC8114043

[2] V. B. Duthon , C. Barea , S. Abrassart , J. H. Fasel , D. Fritschy , and J. Ménétrey , “Anatomy of the Anterior Cruciate Ligament,” Knee Surgery, Sports Traumatology, Arthroscopy 14, no. 3 (2006): 204–213. 10.1007/s00167-005-0679-9 16235056

[3] A. M. Rivera‐Brown , W. R. Frontera , R. Fontánez , and W. F. Micheo , “Evidence for Isokinetic and Functional Testing in Return to Sport Decisions Following ACL Surgery,” PM & R: The Journal of Injury, Function, and Rehabilitation 14, no. 5 (2022): 678–690. 10.1002/pmrj.12815 35411690

[4] J. Chahla , K. N. Kunze , R. F. LaPrade , et al., “The Posteromedial Corner of the Knee: An International Expert Consensus Statement on Diagnosis, Classification, Treatment, and Rehabilitation,” Knee Surgery, Sports Traumatology, Arthroscopy 29, no. 9 (2021): 2976–2986. 10.1007/s00167-020-06336-3 PMC7586411 33104867

[5] J. M. DeLong and B. R. Waterman , “Surgical Techniques for the Reconstruction of Medial Collateral Ligament and Posteromedial Corner Injuries of the Knee: A Systematic Review,” Arthroscopy 31, no. 11 (2015): 2258–2272. 26194939 10.1016/j.arthro.2015.05.011

[6] R. D'Ambrosi , K. Corona , G. Guerra , M. Rubino , F. di Feo , and N. Ursino , “Biomechanics of the Posterior Oblique Ligament of the Knee,” Clinical Biomechanics (Bristol, Avon) 80 (2020): 105205. 33158574 10.1016/j.clinbiomech.2020.105205

[7] R. B. Lundquist , G. R. Matcuk, Jr. , A. J. Schein , et al., “Posteromedial Corner of the Knee: The Neglected Corner,” Radiographics 35, no. 4 (2015): 1123–1137. 26172356 10.1148/rg.2015140166

[8] T. Saigo , G. Tajima , S. Kikuchi , et al., “Morphology of the Insertions of the Superficial Medial Collateral Ligament and Posterior Oblique Ligament Using 3‐Dimensional Computed Tomography: A Cadaveric Study,” Arthroscopy 33, no. 2 (2017): 400–407. 27780652 10.1016/j.arthro.2016.07.030

[9] T. D. Vieira , C. Pioger , F. Franck , et al., “Arthroscopic Dissection of the Distal Semimembranosus Tendon: An Anatomical Perspective on Posteromedial Instability and Ramp Lesions,” Arthroscopy Techniques 8, no. 9 (2019): e987–e991. 31687330 10.1016/j.eats.2019.05.008 PMC6819869

[10] C. Kittl , D. K. Becker , M. J. Raschke , et al., “Dynamic Restraints of the Medial Side of the Knee: The Semimembranosus Corner Revisited,” American Journal of Sports Medicine 47, no. 4 (2019): 863–869. 30870030 10.1177/0363546519829384

[11] R. D'Ambrosi , K. Corona , G. Guerra , et al., “Midterm Outcomes, Complications, and Return to Sports After Medial Collateral Ligament and Posterior Oblique Ligament Reconstruction for Medial Knee Instability: A Systematic Review,” Orthopaedic Journal of Sports Medicine 9, no. 11 (2021): 23259671211056070. 34888393 10.1177/23259671211056070 PMC8649099

[12] R. D'Ambrosi , L. M. Sconfienza , D. Albano , et al., “High Incidence of RAMP Lesions and a Nonnegligible Incidence of Anterolateral Ligament and Posterior Oblique Ligament Rupture in Acute ACL Injury,” Knee Surgery, Sports Traumatology, Arthroscopy 32, no. 8 (2024): 1992–2002. 10.1002/ksa.12219 38686571

[13] F. Vosoughi , R. Rezaei Dogahe , A. Nuri , M. Ayati Firoozabadi , and J. Mortazavi , “Medial Collateral Ligament Injury of the Knee: A Review on Current Concept and Management,” Archives of Bone and Joint Surgery 9, no. 3 (2021): 255–262. 34239952 10.22038/abjs.2021.48458.2401 PMC8221433

[14] R. F. Laprade and C. A. Wijdicks , “The Management of Injuries to the Medial Side of the Knee,” Journal of Orthopaedic and Sports Physical Therapy 42, no. 3 (2012): 221–233. 22382986 10.2519/jospt.2012.3624

[15] M. E. Cinque , J. Chahla , B. M. Kruckeberg , N. N. DePhillipo , G. Moatshe , and R. F. LaPrade , “Posteromedial Corner Knee Injuries: Diagnosis, Management, and Outcomes: A Critical Analysis Review,” JBJS Review 5, no. 11 (2017): e4. 10.2106/JBJS.RVW.17.00004 29200405

[16] J. A. Braaten , M. T. Banovetz , A. N. Rodriguez , P. Thomas , and R. LaPrade , “From Anatomy to Complex Reconstruction: A Modern Review on the Medial Collateral Ligament of the Knee,” Archives of Bone and Joint Surgery 10, no. 10 (2022): 818–826. 36452420 10.22038/ABJS.2022.66697.3179 PMC9702019

[17] X. Liu , H. Feng , H. Zhang , et al., “Surgical Treatment of Subacute and Chronic Valgus Instability in Multiligament‐Injured Knees With Superficial Medial Collateral Ligament Reconstruction Using Achilles Allografts: A Quantitative Analysis With a Minimum 2‐Year Follow‐Up,” American Journal of Sports Medicine 41, no. 5 (2013): 1044–1050. 23467556 10.1177/0363546513479016

[18] J. D. Hughes , T. Rauer , C. M. Gibbs , and V. Musahl , “Diagnosis and Treatment of Rotatory Knee Instability,” Journal of Experimental Orthopaedics 6, no. 1 (2019): 48. 31865518 10.1186/s40634-019-0217-1 PMC6925612

[19] P. Kyritsis , R. Bahr , P. Landreau , R. Miladi , and E. Witvrouw , “Likelihood of ACL Graft Rupture: Not Meeting Six Clinical Discharge Criteria Before Return to Sport Is Associated With a Four Times Greater Risk of Rupture,” British Journal of Sports Medicine 50, no. 15 (2016): 946–951. 27215935 10.1136/bjsports-2015-095908

[20] S. A. Herring , W. B. Kibler , and M. Putukian , “The Team Physician and the Return‐To‐Play Decision: A Consensus Statement‐2012 Update,” Medicine and Science in Sports and Exercise 44, no. 12 (2012): 2446–2448. 23160348 10.1249/MSS.0b013e3182750534

[21] N. van Melick , R. E. H. van Cingel , F. Brooijmans , et al., “Evidence‐Based Clinical Practice Update: Practice Guidelines for Anterior Cruciate Ligament Rehabilitation Based on a Systematic Review and Multidisciplinary Consensus,” British Journal of Sports Medicine 50, no. 24 (2016): 1506–1515. 27539507 10.1136/bjsports-2015-095898

[22] L. Engebretsen and M. Lind , “Anteromedial Rotatory Laxity,” Knee Surgery, Sports Traumatology, Arthroscopy 23, no. 10 (2015): 2797–2804. 10.1007/s00167-015-3675-8 26085190

[23] R. D'Ambrosi , K. Corona , G. Guerra , et al., “Posterior Oblique Ligament of the Knee: State of the Art,” EFORT Open Review 6, no. 5 (2021): 364–371. 10.1302/2058-5241.6.200127 PMC8183151 34150330

[24] J. E. Rayes , T. Fradin , C. Ngbilo , et al., “Posterior Oblique Ligament Repair Concomitant to Anterior Cruciate Ligament Reconstruction,” Arthroscopy Techniques 10, no. 2 (2021): e551–e554. 33680791 10.1016/j.eats.2020.10.037 PMC7917300

[25] B. Kerzner , H. W. Swindell , E. B. Terhune , et al., “Medial Collateral Ligament and Posterior Oblique Ligament Reconstruction for Valgus Instability After Total Knee Arthroplasty,” Arthroscopy Techniques 11, no. 9 (2022): e1531–e1539. 36185120 10.1016/j.eats.2022.04.003 PMC9519797

[26] S. Fusco , D. Albano , S. Gitto , F. Serpi , C. Messina , and L. M. Sconfienza , “Posteromedial Corner Injuries of the Knee: Imaging Findings,” Seminars in Musculoskeletal Radiology 28, no. 3 (2024): 318–326. 38768596 10.1055/s-0044-1779718

[27] A. Deichsel , C. Peez , M. J. Raschke , et al., “A Flat Reconstruction of the Medial Collateral Ligament and Anteromedial Structures Restores Native Knee Kinematics: A Biomechanical Robotic Investigation,” American Journal of Sports Medicine 52, no. 13 (2024): 3306–3313. 39360333 10.1177/03635465241280984 PMC11542325

[28] S. Ball , J. M. Stephen , H. el‐Daou , A. Williams , and A. A. Amis , “The Medial Ligaments and the ACL Restrain Anteromedial Laxity of the Knee,” Knee Surgery, Sports Traumatology, Arthroscopy 28, no. 12 (2020): 3700–3708. 10.1007/s00167-020-06084-4 PMC7669770 32504158

[29] E. Herbst , R. J. Muhmann , M. J. Raschke , et al., “The Anterior Fibers of the Superficial MCL and the ACL Restrain Anteromedial Rotatory Instability,” American Journal of Sports Medicine 51, no. 11 (2023): 2928–2935. 37503921 10.1177/03635465231187043

[30] Q. He , Q. Liang , and H. Zhang , “Effect of Posterior Oblique Ligament Repair on Rotational Stability of Knee Joint,” Zhongguo Xiu Fu Chong Jian Wai Ke Za Zhi 33, no. 5 (2019): 551–554. 31090347 10.7507/1002-1892.201810012 PMC8337201

[31] J. Li , J. Tang , L. Yao , et al., “The Validity of the Ligs Digital Arthrometer at Different Loads to Evaluate Complete ACL Ruptures,” Frontiers in Bioengineering and Biotechnology 11 (2023): 1049100. 36998807 10.3389/fbioe.2023.1049100 PMC10046814

[32] D. Wu , D. Wang , Y. Han , L. Guo , and S. Wang , “A Novel Digital Arthrometer to Measure Anterior Tibial Translation,” Journal of Orthopaedic Surgery and Research 18, no. 1 (2023): 101. 36782204 10.1186/s13018-022-03497-4 PMC9926554

[33] F. R. Noyes , E. S. Grood , and P. A. Torzilli , “Current Concepts Review. The Definitions of Terms for Motion and Position of the Knee and Injuries of the Ligaments,” Journal of Bone and Joint Surgery. American Volume 71, no. 3 (1989): 465–472. 2647751

[34] F. R. Noyes , S. F. Stowers , E. S. Grood , J. Cummings , and L. A. VanGinkel , “Posterior Subluxations of the Medial and Lateral Tibiofemoral Compartments. An In Vitro Ligament Sectioning Study in Cadaveric Knees,” American Journal of Sports Medicine 21, no. 3 (1993): 407–414. 8346756 10.1177/036354659302100314

[35] D. E. Cooper , “Tests for Posterolateral Instability of the Knee in Normal Subjects. Results of Examination Under Anesthesia,” Journal of Bone and Joint Surgery. American Volume 73, no. 1 (1991): 30–36. 1985992

[36] C. J. Griffith , R. F. LaPrade , S. Johansen , B. Armitage , C. Wijdicks , and L. Engebretsen , “Medial Knee Injury: Part 1, Static Function of the Individual Components of the Main Medial Knee Structures,” American Journal of Sports Medicine 37, no. 9 (2009): 1762–1770. 19609008 10.1177/0363546509333852

