# Supplementary material for: Clinical Effect of Posterior Obliqueligament Repair in Anterior Cruciate Ligament Reconstruction Combined With Medial Collateral Ligament Repair: A Retrospective Comparative Study
Source: J Cell Mol Med. 2025 Jul 24;29(14):e70690. doi: 10.1111/jcmm.70690 (PMC12287616; doi:10.1111/jcmm.70690)
Supplement: Supplementary file 1 — Appendix S1. [file JCMM-29-e70690-s001.docx]

Materials Design Analysis Reporting (MDAR)

Checklist for Authors

The MDAR checklist is a tool for authors, editors and others seeking to adopt the MDAR framework for transparent reporting in manuscripts and other outputs. Please refer to the MDAR Elaboration Document for additional context for the MDAR framework.

Materials

| **Antibodies** | **Yes (indicate where provided: section/paragraph)** | **n/a** |
| --- | --- | --- |
| For commercial reagents, provide supplier name, catalogue number and RRID, if available. |  | N/A |
|  |  |  |
| **Cell materials** | **Yes (indicate where provided: section/paragraph)** | **n/a** |
| **Cell lines:** Provide species information, strain. Provide accession number in repository **OR** supplier name, catalog number, clone number, **OR** RRID |  | N/A |
| **Primary cultures:** Provide species, strain, sex of origin, genetic modification status. |  | N/A |
|  |  |  |
| **Experimental animals** | **Yes (indicate where provided: section/paragraph)** | **n/a** |
| **Laboratory animals:** Provide species, strain, sex, age, genetic modification status. Provide accession number in repository **OR** supplier name, catalog number, clone number, **OR** RRID |  | N/A |
| **Animal observed in or captured from the field:** Provide species, sex and age where possible |  | N/A |
| **Model organisms:** Provide Accession number in repository (where relevant) **OR** RRID |  | N/A |
|  |  |  |
| **Plants and microbes** | **Yes (indicate where provided: section/paragraph)** | **n/a** |
| **Plants:** provide species and strain, unique accession number if available, and source (including location for collected wild specimens) |  | N/A |
| **Microbes:** provide species and strain, unique accession number if available, and source |  | N/A |
|  |  |  |
| **Human research participants** | **Yes (indicate where provided: section/paragraph)** | **n/a** |
| Identify authority granting ethics approval (IRB or equivalent committee(s), provide reference number for approval. | Yes, Ethics Committee of Ordos Central Hospital(2024-124) |  |
| Provide statement confirming informed consent obtained from study participants. | Yes |  |
| Report on age and sex for all study participants. | Yes |  |

Design

| **Study protocol** | **Yes (indicate where provided: section/paragraph)** | **n/a** |
| --- | --- | --- |
| For clinical trials, provide the trial registration number **OR** cite DOI in manuscript. |  | N/A |
|  |  |  |
| **Laboratory protocol** | **Yes (indicate where provided: section/paragraph)** | **n/a** |
| Provide DOI or other citation details if detailed step-by-step protocols are available. |  | N/A |
|  |  |  |
| **Experimental study design (statistics details)** | **Yes (indicate where provided: section/paragraph)** | **n/a** |
| State whether and how the following have been done**, or** if they were not carried out. | Yes |  |
| Sample size determination | Materials and methods (Paragraph 8/Line 243-248) |  |
| Randomisation | Materials and methods (Paragraph 7/Line 206-208) |  |
| Blinding | Materials and methods (Paragraph 7/Line 209-210) |  |
| Inclusion/exclusion criteria | Materials and methods (Paragraph 4/Line 122-136) |  |
|  |  |  |
| Sample definition and in-laboratory replication | **Yes (indicate where provided: section/paragraph)** | **n/a** |
| State number of times the experiment was replicated in laboratory |  |  |
| Define whether data describe technical or biological replicates |  |  |
|  |  |  |
| Ethics | **Yes (indicate where provided: section/paragraph)** | **n/a** |
| Studies involving human participants: State details of authority granting ethics approval (IRB or equivalent committee(s), provide reference number for approval. | Yes, Ethics Committee of Ordos Central Hospital(2024-124) |  |
| Studies involving experimental animals: State details of authority granting ethics approval (IRB or equivalent committee(s), provide reference number for approval. |  | N/A |
| Studies involving specimen and field samples: State if relevant permits obtained, provide details of authority approving study; if none were required, explain why. |  | N/A |
|  |  |  |
| Dual Use Research of Concern (DURC) | **Yes (indicate where provided: section/paragraph)** | **n/a** |
| If study is subject to dual use research of concern, state the authority granting approval and reference number for the regulatory approval |  | N/A |

Analysis

| **Attrition** | **Yes (indicate where provided: section/paragraph)** | **n/a** |
| --- | --- | --- |
| State if sample or data point from the analysis is excluded, and whether the criteria for exclusion were determined and specified in advance. | Yes |  |
|  |  |  |
| **Statistics** | **Yes (indicate where provided: section/paragraph)** | **n/a** |
| Describe statistical tests used and justify choice of tests. | Baseline characteristics will be compared using T-tests, Chi-square tests, or rank sum tests.  Multivariate covariance analysis |  |
|  |  |  |
| **Data Availability** | **Yes (indicate where provided: section/paragraph)** | **n/a** |
| State whether newly created datasets are available, including protocols for access or restriction on access. |  | N/A |
| If data are publicly available, provide accession number in repository or DOI or URL. |  | N/A |
| If publicly available data are reused, provide accession number in repository or DOI or URL, where possible. |  |  |
|  |  |  |
| **Code Availability** | **Yes (indicate where provided: section/paragraph)** | **n/a** |
| For all newly generated code and software essential for replicating the main findings of the study: |  | N/A |
| State whether the code or software is available. |  | N/A |
| If code is publicly available, provide accession number in repository, or DOI or URL. |  | N/A |

Reporting

| **Adherence to community standards** | **Yes (indicate where provided: section/paragraph)** | **n/a** |
| --- | --- | --- |
| MDAR framework recommends adoption of discipline-specific guidelines, established and endorsed through community initiatives. Journals have their own policy about requiring specific guidelines and recommendations to complement MDAR. |  | N/A |
| State if relevant guidelines (eg., ICMJE, MIBBI, ARRIVE) have been followed, and whether a checklist (eg., CONSORT, PRISMA, ARRIVE) is provided with the manuscript. | ICMJE guidelines were followed, as the journal follows ICMJE recommendations for publication.  (Please do not delete this sentence) |  |

| Please leave this space alone as it will be supplemented by the editorial office when needed. |
| --- |
