# Supplementary figures and images for: Clinical Effect of Posterior Obliqueligament Repair in Anterior Cruciate Ligament Reconstruction Combined With Medial Collateral Ligament Repair: A Retrospective Comparative Study
Source: J Cell Mol Med. 2025 Jul 24;29(14):e70690. doi: 10.1111/jcmm.70690 (PMC12287616; doi:10.1111/jcmm.70690)

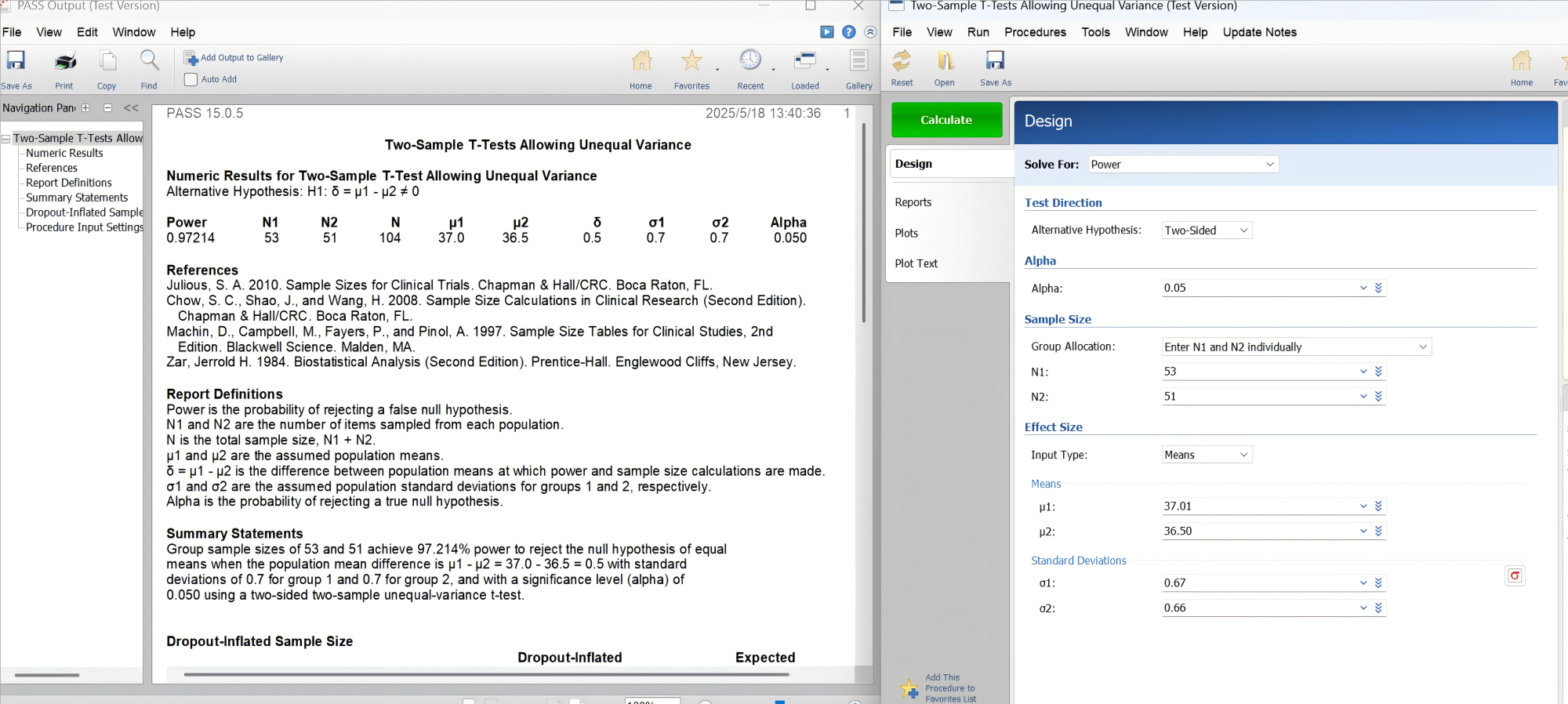

Supplement: Supplementary file 2 — Appendix S2. [file JCMM-29-e70690-s003.png]
